# Characteristics of human primary mantle cell lymphoma engraftment in NSG mice

**DOI:** 10.1111/bjh.13581

**Published:** 2015-07-28

**Authors:** Sunil Iyengar, Linda Ariza‐McNaughton, Andrew Clear, David Taussig, Rebecca Auer, Amy Roe, Debra Lillington, Sameena Iqbal, Simon Joel, John Gribben, Dominique Bonnet

**Affiliations:** ^1^ Department of Haemato‐OncologyRoyal Marsden HospitalSuttonUK; ^2^ Barts Cancer InstituteQueen Mary University of LondonCharterhouse SquareLondonUK; ^3^ Haematopoeitic stem cell laboratoryThe Francis Crick InstituteLincoln's Inn FieldsLondonUK; ^4^ Cytogenetics DepartmentBarts Health NHS TrustThe Royal London HospitalLondonUK

**Keywords:** mantle cell lines, mantle cell primary samples, xenotransplantation, intravenous injection

Mantle cell lymphoma (MCL) is a clinically heterogeneous, but often aggressive lymphoma characterized by the *IGH:CCND1* translocation and cyclin D1 (CCND1) over‐expression. Chromosomal instability, due to disrupted DNA damage response, in conjunction with abnormal activation of cell survival mechanisms underlies the aggressive clinical course in MCL (Jares *et al*, 2012).

In recent years, improved understanding of lymphoma biology has led to the development of a number of small molecule inhibitors. However, the relative rarity of MCL (incidence 0·55 per 100 000) (Smedby & Hjalgrim, 2011) poses a challenge in effectively evaluating these drugs in patients.

*In vitro* studies have been limited by the difficulty of culturing primary MCL cells. Murine models of MCL cell lines are relatively easy to establish in SCID or NOD/SCID/IL2Rγ null (NSG) mice (Wang *et al*, 2007, 2008a; Weston *et al*, 2010) but have their limitations. Until a couple of years ago, the only primary mouse model of human MCL described in the literature was established by injection of primary MCL cells into subcutaneous human bone grafts implanted in SCID mice (SCID‐Hu model) (Wang *et al*, 2008b). Recently, however, disseminated models of human primary MCL have been established in NSG mice (Iyengar *et al*, 2012; Klanova *et al*, 2014). We report our experience here in further detail, focussing on the characteristics of MCL engraftment in this model.

We used 8‐ to 12‐week‐old NSG mice that were sub‐lethally irradiated (3·75 Gy) 24 h prior to transplantation. Before undertaking xenograft studies with primary cells, we used the MCL cell line JEKO‐1 to assess kinetics, disease burden and distribution of MCL cells in NSG mice. JEKO‐1 cells were transduced with firefly luciferase and injected intravenously into irradiated mice at two doses – 0·5 × 10^6^ and 2 × 10^6^ cells. Bioluminescent imaging was performed at weekly intervals following injection of D‐luciferin. All mice became ill with marked weight loss and had to be sacrificed by day 29. Bioluminescence was observed in the bone marrow and spleen in all mice. Mice injected with the higher cell dose had more rapid disease progression, developed hind leg weakness and had bioluminescence in the central nervous system (CNS) on imaging, indicating involvement (Fig 1).

**Figure 1:**
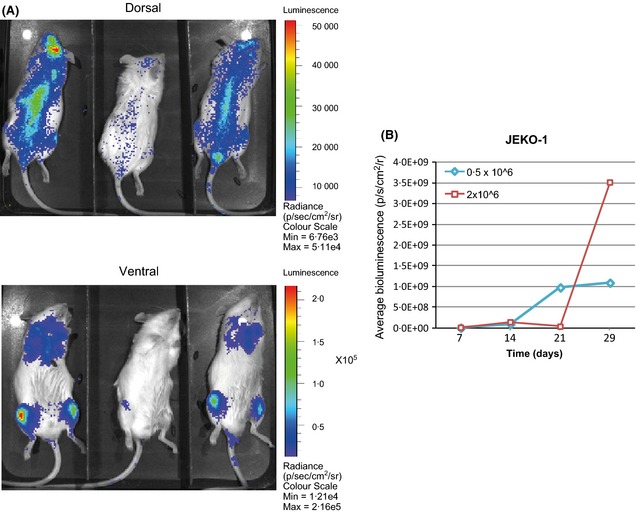
Longitudinal quantitative analysis of bioluminescent imaging (BLI): NSG mice were injected intravenously with the JEKO‐1 cell line transduced with luciferase (Luc) reporter constructs. (A) Representative dorsal view (upper panels) and ventral view (lower panels) of BLI kinetics in three mouse injected with control Luc^+^ transduced JEKO‐1 cells on Day 7 (2 × 10^6^ cells) (B). Graph comparing average increase in bioluminescence over 4 weeks between mice injected with 0·5 × 10^6^ cells (blue) and those injected with 2 × 10^6^ cells (red). A steep increase in bioluminescence was observed after Day 21 in mice injected with the higher concentration (2 × 10^6^ cells) of JEKO‐1 cells. Bioluminescence was measured as photons per second per square centimetre per radian (p/s/cm^2^/r, FLUX), of JEKO‐1 Luc^+^ cells and was quantified at the indicated time points. The values are the mean of the sum of measured signal from both ventral and dorsal positions at each time point.

Following this, seven cryopreserved primary MCL samples were identified from the Barts Cancer Institute tissue bank. An additional fresh primary sample derived from a splenectomy was included in the cohort. Ethical approval was obtained from East London and the City Local Research Ethics Committee. Written informed consent was obtained from patients according to the Declaration of Helsinki. All samples had a classical MCL phenotype with CD5/CD20 positivity and were confirmed to have the *IGH:CCND1* translocation by fluorescence *in situ* hybridization (FISH). Irradiated NSG mice were injected intravenously with a dose of 10^7^ unselected MCL cells each. Flow cytometry was performed for mouse CD45 and human CD45, CD3, CD5 and CD20 on peripheral blood samples taken from mice at 3, 6 and 12 weeks. Mice were sacrificed at 20 weeks, or earlier if they met Home Office guidelines, and tissue was harvested for immunohistochemistry (IHC). Cells were flushed from mouse femur for flow cytometry.

At 20 weeks, MCL cells were found in the bone marrow and spleen of mice injected with 2 out of the 7 cryopreserved primary samples. Both samples that engrafted had blastoid morphology and one was obtained from a patient with relapsed disease. FISH for *IGH:CCND1* on cell suspensions prepared from spleen of NSG mice further confirmed engraftment. None of the mice that engrafted appeared to have bowel involvement as assessed by IHC. Lymphadenopathy was not found at sacrifice. Scattered human CD20‐positive cells were seen in the liver but this was not a consistent feature. Mice remained relatively well until sacrifice (Fig 2A–F). As a next step, secondary transplantation of MCL cells isolated from NSG spleen (10^7^ cells per mouse) was undertaken. Once again, engraftment was seen in mouse spleen and bone marrow on sacrifice at 20 weeks (Fig 2G).

**Figure 2:**
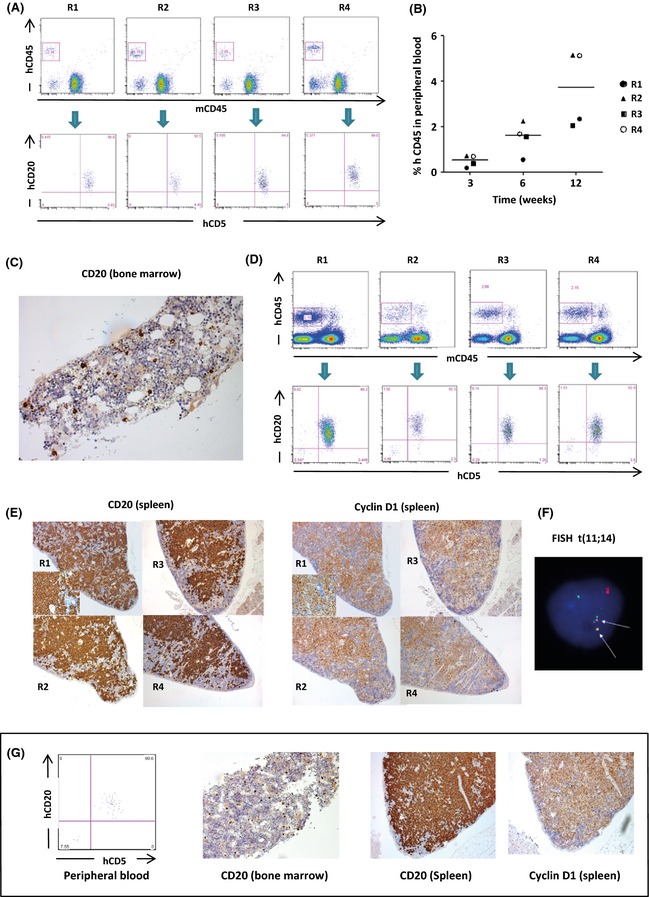
Engraftment of human primary mantle cell lymphoma (MCL) in NSG mice: T‐cell depleted human primary mononuclear cells were injected intravenously at a dose of 10^7^ cells per mouse. Peripheral blood was sampled from mice at 3, 6 and 12 weeks, followed by sacrifice at 20 weeks. At 20 weeks, Bone marrow and spleen sections were used for immunohistochemistry and spleen cells were isolated for Fluorescence *in situ* hybridization (FISH) analysis [t(11; 14)] (Replicate mice labelled R1 to R4). (A) Flow cytometric analysis of peripheral blood at 12 weeks showing human (h)CD45^+^ cells that co‐expressed hCD20 and hCD5 in 4 replicate mice. (B) Dot plot showing the percentage of hCD45^+^ cells per mouse overtime. Lines represent mean percentage of hCD45^+^ cells. (C) Scattered hCD20^+^ cells seen on immunohistochemical staining of decalcified, paraffin‐embedded femoral bone marrow section (original magnification ×400). (D) Flow cytometric analysis of the bone marrow at 20 weeks showing hCD45^+^ cells that co‐expressed hCD20 and hCD5 in the same mice. (E) Images of hCD20 expression and cyclin D1 immunohistochemistry on formalin‐fixed, paraffin‐embedded splenic sections from mice showing heavy infiltration of hCD20 and cyclin D1 staining cells (original magnification: ×100). The inserts show hCD20 and cyclin D1 at higher magnification (×200). (F) Abbott Molecular IGH‐CCND1 dual colour, dual fusion translocation probe demonstrating abnormal t(11;14) fusion signal pattern (indicated by arrows) in cells isolated from mouse spleen. *IGH* (14q32) = Spectrum Green, *CCND1* (11q13) = Spectrum Orange. (G) Unselected splenic cells isolated from mice in the preceding experiment were injected into 3 mice (10^7^ cells per mouse) and engraftment was seen in 2 out 3 mice. Representative images showing secondary engraftment of MCL in peripheral blood (12 weeks), bone marrow (20 weeks) and spleen (20 weeks) of NSG mice.

In addition to the two cryopreserved samples, evidence of engraftment was also seen in the spleen of NSG mice injected with the fresh primary sample (non‐blastoid). Interestingly, there appeared to be co‐existence of MCL cells and T‐cells in the spleen of mice injected with fresh MCL cells, with tumour cells concentrated around blood vessels. However, these mice had to be sacrificed at 7 weeks due to illness and T‐cell infiltration was found in the liver and bone marrow, without evidence of MCL. We found a similar proliferation of T cells but without evidence of MCL in one of the seven cryopreserved samples that had high T‐cell content (>10%), indicating T‐cell depletion may be important in this scenario.

Therefore, similar to the recent report by Klanova *et al* (2014), we demonstrate human primary MCL engraftment in NSG mice. In contrast to their study where mice were injected with a variable cell dose (1–8 × 10^7^ cells), we injected all mice with a fixed dose of 10^7^ cells. This may explain the lower rate of engraftment in our study. Both cryopreserved samples that engrafted in our study had blastoid morphology, suggesting that a higher cell dose may be required for engraftment of non‐blastoid MCL in this model.

In our experiments, mice with primary MCL engraftment were not visibly ill at 20 weeks and disease burden was heaviest in the spleen. In contrast, disease progression was rapid in the JEKO‐1 xenograft, with CNS involvement and hind leg weakness developing by 4 weeks. These findings mirror those of Klanova *et al* (2014)*,* and are important considerations when designing pre‐clinical experiments involving these models. The longer overall survival of NSG mouse models of primary human MCL could be an advantage for pre‐clinical testing of newer agents, which often require longer periods of administration for efficacy.

Finally, our study demonstrates, similar to the findings of Klanova *et al* (2014)*,* that secondary transplantation can be successfully carried out in this model, highlighting the self‐renewal and tumour‐initiating capacity of primary MCL cells. In summary, this NSG model of human primary MCL is a promising *in vivo* model for both pre‐clinical drug testing and further understanding MCL biology. Our research provides further insight into the advantages and limitations of this model, which will be crucial for its effective use in pre‐clinical research.

## Author contributions

S.I.: design and performance of experiments, data analysis and interpretation, manuscript writing; L.A.M.: design and performance of experiments, data analysis and interpretation; A.C. and S.I.: help in sample processing, research and data analysis; R.A., D.T. and J.G.: provision of vital patient samples, materials and data; A.R. and D.L. performance of FISH analysis; D.B.: design of experiments, data analysis, manuscript writing.

## Financial disclosure

The authors declare no potential conflicts of interest.
